# Phase-II Trials of Pazopanib in Metastatic Neuroendocrine Neoplasia (mNEN): A Systematic Review and Meta-Analysis

**DOI:** 10.3389/fonc.2020.00414

**Published:** 2020-04-07

**Authors:** Alberto Bongiovanni, Chiara Liverani, Federica Recine, Valentina Fausti, Laura Mercatali, Alessandro Vagheggini, Chiara Spadazzi, Giacomo Miserocchi, Claudia Cocchi, Giandomenico Di Menna, Alessandro De Vita, Stefano Severi, Silvia Nicolini, Toni Ibrahim

**Affiliations:** ^1^Osteoncology and Rare Tumors Center, Istituto Scientifico Romagnolo per lo Studio e la Cura dei Tumori (IRST) IRCCS, Meldola, Italy; ^2^Unit of Biostatistics and Clinical Trials, Istituto Scientifico Romagnolo per lo Studio e la Cura dei Tumori (IRST) IRCCS, Meldola, Italy; ^3^Nuclear Medicine Unit, Istituto Scientifico Romagnolo per lo Studio e la Cura dei Tumori (IRST) IRCCS, Meldola, Italy

**Keywords:** pazopanib, neuroendocrine neoplasia, neuroendocrine tumours, review, carcinoid

## Abstract

**Background:** Several phase-II trials have been designed to evaluate tyrosine kinase inhibitors (TKIs), in particular, pazopanib in neuroendocrine neoplasia (NEN), but its efficacy has not yet been demonstrated in a randomised-controlled Phase III trial. A systematic review of the published clinical trials of metastatic NEN patients could reduce the possible bias of single phase II studies. The present systematic review focuses on the efficacy and safety of pazopanib in patients with metastatic and locally advanced NEN.

**Methods:** A systematic search in the major databases Medline/PubMed, Cochrane and Embase and in supplementary material from important international Meetings was performed to identify publications on pazopanib for the treatment of neuroendocrine neoplasia. English language was defined as a restriction. Four authors of the present review independently performed the study selection, assessed the risk of bias and extracted study data. Four published clinical trials and 2 abstracts were identified. One trial was excluded because the topic was Von-Hippel Landau disease and one abstract was eliminated because of the lack of information on meeting proceedings.

**Results:** In all of the trials pazopanib was orally administered at a dose of 800 mg daily continuously with a 28-day cycle. The intention-to-treat population for efficacy was composed of 230 patients with a median age of 62 years. The partial response rate was 10.7% (95% confidence interval 2.6–20.5). The rate for stable disease was 79.6% (range: 61.7–92.1%) with a disease control rate (DCR) of 90.3%. Progressive disease was reported in 9.7% (range 5.2–17.6) of patients. No complete responses were observed. Median progression-free survival was 11.6 months (95% CI: 9.2–13.9). Overall survival from all the trials was 24.6 (95% CI: 18.7–40.8) months. Severe adverse events (grade III–IV) included hypertension 31%, 16% increase in AST/ALT, diarrhoea 10% and fatigue 10%.

**Conclusions:** Pazopanib monotherapy achieved a DCR of 90.3% in patients with locally advanced and/or metastatic neuroendocrine neoplasia, with an overall response rate comparable to other TKIs and mTOR inhibitors and a safety profile similar to that of drugs of the same class.

## Introduction

### Rationale

Lung and gastroenteropancreatic (GEP) neuroendocrine tumours (NETs) are a heterogeneous group of malignancies derived from neuroendocrine cell compartments in various organs (1). A significant increase in the incidence of NETs over time has been reported ranging from 2.5 to 5 cases per 100,000 in Caucasian population (2–5). In unresectable or metastatic NETs, systemic treatment options are limited but in recent years there has been a renewed interest in expanding the therapeutic armamentarium (6). In particular, whilst in GEP-NETs the activity and safety of several compounds has been explored, in lung NETs only few drugs have been tested and the choice of treatment is often based on GEP-NET studies (7, 8).

NETs have been identified as hypervascular tumours. Vascular endothelial growth factor (VEGF) and VEGF receptors (VEGFRs) are usually overexpressed and are associated with poor prognosis (9). However, a modest clinical activity with bevacizumab, a monoclonal antibody targeting VEGF, has been observed in advanced neuroendocrine tumours in phase II studies (10, 11). In a phase III trial, sunitinib showed a superior efficacy to placebo in terms of progression-free survival (PFS) (11.4 vs. 5.5 months) leading to FDA and EMA approval for use in patients with advanced pancreatic NETs (pNETs) (12).

Pazopanib is an oral multitargeted tyrosine kinase inhibitor acting through VEGFR types 1–3, fibroblast-derived growth factor receptors (FGFR 1, 3, and 4), platelet-derived growth factor receptors α and β, and stem-cell factor receptor (c-Kit) (13, 14). Studies *in vitro* have shown that pazopanib inhibits ligand-induced autophosphorylation of VEGFR-2 PDGF-induced phosphorylation of c-Kit and PDGFRβ and VEGF-induced proliferation (13). *In vivo* pazopanib is known to inhibit FGF- and VEGF-induced angiogenesis in mouse models and has shown antitumour activity in different human models of solid tumours (15).

In one phase I trial, a patient with unknown primary neuroendocrine tumour obtained a partial response (PR) from treatment with pazopanib (16). Nevertheless, there are limited and non-conclusive data on the efficacy of tyrosine kinase inhibitors (TKIs) in both pNETs and non-pNETs, especially in those originating from the colorectum and small intestine where the incidence of the disease is high (6, 17).

### Objectives

The aim of this systematic review was to evaluate the published studies assessing the activity and safety of pazopanib in patients with metastatic NEN (mNEN).

### Research Questions

- Activity of pazopanib in patients with mNEN
- Safety of pazopanib in patients with mNEN
- Role of pazopanib in the therapeutic scenario of mNEN.

## Methods

### Study Design

We report the results of a phase II systematic review and meta-analysis on the activity and safety of pazopanib in patients with mNEN. This study was performed according to PRISMA guidelines (18, 19)(see Supplementary Materials). The quality of included studies was assessed using the Downs and Black checklist (D&B checklist), which is appropriate for both randomised and non-randomised clinical trials. This checklist consists of 27 items distributed between five subscales. The total maximum score is 32. A study scoring 16 or more is ranked as a high quality study (20).

### Participants, Interventions, Comparator

We included all articles with prospective data on mNEN in adult patients treated with pazopanib. All of the studies included were in the English language.

### Systematic Review Protocol

We developed a protocol that had pre-specified objectives, eligibility criteria, data of interest, search strategy, and analysis plan. The present systematic review was registered in the PROSPERO database.

### Data Source Study Section and Data Extraction

**Figure 1:**
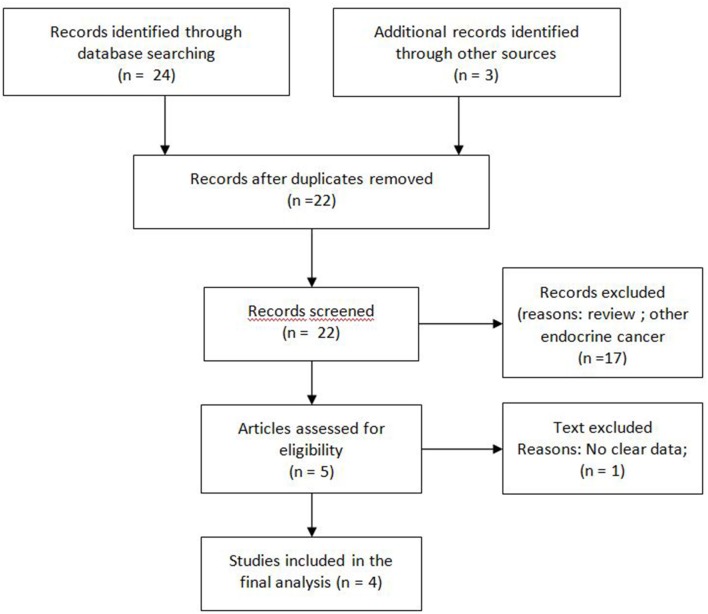
Flow diagram of search methods.

A search of the major databases Medline/PubMed, Cochrane and Embase was performed to identify publications on pazopanib for the treatment of neuroendocrine neoplasia (21). Search terms used included “pazopanib” and/or “neuroendocrine.” A supplementary search of congress abstracts published between 2014 and 2019 was also carried out for the annual meetings of the American Society of Clinical Oncology (ASCO), ASCO Gastrointestinal Symposium (ASCO-GI), and European Society for Medical Oncology (ESMO). A manual search of the references of retrieved articles for additional relevant publications was also performed. References from systematic reviews and meta-analyses were screened to ensure search sensitivity (Figure 1).

Two authors independently conducted a preliminary screening of reports by reading titles and abstracts. Duplicate publications were removed. All identified citations were reviewed and those considered unrelated were excluded. The full texts of potentially relevant articles were then downloaded for the second round of screening. When disagreement existed, two authors discussed with a third reviewer to reach a final decision. Data from included studies describing the population treated as well as treatment efficacy and toxicity parameters were extracted and pooled.

For each study, the following data were collected and tabularised for the analysis: year of publication, name of the first author, area of study; study design; baseline characteristics of patients included; intervention including regimens, dosages and cycles; outcomes including overall response rate (ORR), disease control rate (DCR), progression-free survival (PFS) and overall survival (OS); toxicities including those of a haematological and non-haematological nature.

### Statistical Analysis

For survival primary endpoints, meta-analyses usually deal with hazard ratios which can only be obtained when the experimental treatment is compared to a control treatment. However, single-arm exploratory phase II studies aimed at estimating the survival curve are far from rare, especially in the area of rare tumours. In this scenario, the PFS and OS curves are usually summarised by medians and accompanied by their 95% confidence interval (95% CI), as is the case of the present review. Following the method used by McGrath et al., pooled estimates were obtained as the median of the study-specific PFS and OS medians (22), whereas the corresponding 95%CIs were obtained as the $1/2\pm \min\left\{1/2,\;z_{0.975}/\left(2\sqrt{K}\right)\right\}$ quantiles of the *k* observed study medians, with *z*_α_ the α quantile of the standard normal distribution.

Heterogeneity between the median PFS and OS of studies was evaluated using the *I*^2^ index that quantifies values higher than 50%, indicating sizable heterogeneity. Furthermore, the Cochran *Q*-test was used to infer the null hypothesis between study homogeneity at a significance level α = 0.10.

All of the statistical analyses were performed with the statistical language R version 3.6.1. The metamedian package was used to compute the pooled estimates and their 95% CIs, while the *ad hoc* code was used to compute the *I*^2^ index and infer homogeneity via the Cochran *Q*-test.

## Results

### Study Selection and Characteristics

The systematic search of the literature identified four studies meeting selection criteria (Figure 1): three peer-reviewed journal publications [(23–25) and one conference abstract/poster (24)]. Briefly, one randomised and three non-randomised prospective phase II studies included a total of 304 patients of whom 74 were treated with placebo. Three studies were multicentric and only one was monocentric (23). Two studies had an independent review (23, 26). All the studies were of high quality according to the D&B checklist. Patient number, tumour histology (grade and primitive site), Eastern Cooperative Oncology Group Performance Status (ECOG PS) and other characteristics of each study are shown in Table 1.

**Table 1:** Principal characteristics of the Phase II studies.

**References**	**Country**	**Phase**	**Type of publication**	**Randomised vs. placebo**	**No. of patients**	**Grade**	**Primitive site**	**ECOG PS**	**Biomarker evaluation**	**Setting**
Ahn et al. (23)	Asia	II	Full Text	No	37	G1 G2 G3	Pancreas GI Lung Unknown Other	0–1	No	Metastatic only
Phan et al. (24)	US	II	Full Text	No	52	G1 G2	GI Pancreas	0–1	No	Metastatic/locally advanced
Grande et al. (25)	Europe	II	Full Text	No	44	G1 G2	Pancreas GI Lung Unknown Other	0–1	Yes	Metastatic/locally advanced
Bergsland et al. (26)	US	II	Abstract	Yes	171	G1 G2 UK	Pancreas GI Lung Other	0–1	Yes	Metastatic/locally advanced

*ECOG PS, Eastern Cooperative Oncology Group Performance Status; GI, gastrointestinal*.

### Summary of Findings

#### Population Characteristics

A total of 304 patients were included in the selected trials. Progressive disease during other previous treatment was found at the time of enrolment in 283 (93.1%) patients. Previous therapies included somatostatin analogues (SSA) in 177 (58.2%) patients, other TKIs in 16 (5.2%), everolimus in 25 (8.2%), both TKI and everolimus in 8 (2.6%), chemotherapy in 56 (18.4%), hepatic locoregional treatment in 38 (12.5%) and other non-specified treatments in 19 (6.2%). One hundred fourteen (37.5%) patients had tumours of gastrointestinal (GI) origin, while the remaining (190, 62.5%) had NEN of lung, pancreatic and unknown origin. The majority of patients (76.3%) had grade 1 or 2 NEN and 15 (5%) had grade 3 NEN. Tumour grade was unknown in 58 (18.7%) patients. Seventy patients had a functioning tumour (23%). SSAs were administered together with pazopanib in 230 (75.6%) patients.

### Clinical Outcomes

**Figure 2:**
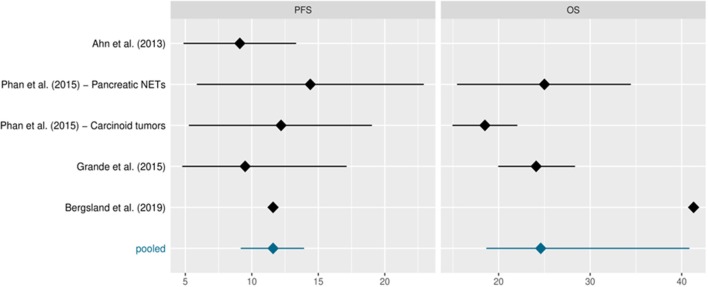
mOS and mPFS in single studies and pooled data. A Forest Plot.

The intention-to-treat population treated with pazopanib comprised 230 patients, excluding 74 patients in the Bergsland study who were treated with placebo. Table 2 shows the study sample sizes or those of the various study arms when reported in the protocol. Median PFS and OS, reported in months, are also included along with their 95%CIs, whenever available. The data derive from single-arm phase II studies, with the exception of Bergsland et al.'s study (26) which was a phase II randomised controlled trial (for the purposes of this review we only considered the experimental pazopanib arm). Phan et al. (24) reported distinct median PFS and OS for patients with pNETs and carcinoid tumours, respectively. Ahn et al. (23) did not evaluate OS and therefore the pooled median was based on the remaining values. Bergsland et al. (26) did not report 95%CIs for PFS or OS. A response to pazopanib was reported in 186 patients. The studies registered stable disease (SD) in 148 (79.5%; range: 95% CI 61.7–92.1%) patients, partial response (PR) in 20 (10.7%; 95% CI, range 2.6–20.5%) and progressive disease (PD) in 18 (9.7%; 95% CI range: 5.8%−17.6%). No complete responses were observed. The DCR was 90.3%. Median PFS and OS from all trials was 11.6 (95% CI: 9.2, 13.9) and 24.6 (95% CI: 18.7, 40.8) months, respectively (Figure 2 and Table 2).

**Table 2:** Sample sizes and median PFS and OS in months along with their 95% confidence intervals (CIs).

**Study**	**Stratification**	**Sample size**	**Median PFS (95% CI)**	**Median OS (95% CI)**
Ahn et al. (23)	–	37	9.1 (4.9, 13.3)	–
Phan et al. (24)	Pancreatic NETs	32	14.4 (5.9, 22.9)	25.0 (15.5, 34.4)
	Carcinoid tumours	20	12.2 (5.3, 19.0)	18.5 (15.0, 22.0)
Grande et al. (25)	–	44	9.5 (4.8, 17.1)	24.1 (20.0, 28.3)
Bergsland et al. (26)	–	97	11.6 (NA, NA)	41.3 (NA, NA)

*PFS, progression-free serviva; OS, overall survival; NA, not applicable*.

### Side-Effects

Safety outcomes are presented in Table 3. The rate of G1-G4 toxicities experienced was 70%. The most frequent adverse events were fatigue (65%), hypertension (50%), neutropoenia (26.5%), mucositis (16%), H&F syndrome (15.6%), thrombocytopoenia (15.2%), anaemia (9.1%) and proteinuria (4.7%). The rate of grade (G)3-4 toxicity was 45.2%. The most frequent G3-G4 adverse event was hypertension (15.6%).

**Table 3:** List of side-effects grouped by grade.

	**Grade 1-2**	**Grade 3-4**	**All grade**
	**no. (%)**	**no. (%)**	**no. (%)**
**Haematological side-effects**			
Anaemia	16 (80.0)	4 (20.0)	20 (100)
Neutropoenia	40 (87.0)	6 (13.0)	46 (100)
Thrombocytopoenia	34 (97.1)	1 (2.9)	35 (100)
**Non-haematological side-effects**			
Abdominal pain	45 (91.8)	4 (8.2)	49 (100)
Alkaline phosphatase	9 (90.0)	1 (10.0)	10 (100)
Alopoecia	7 (100)	0	7 (100)
Anorexia	34 (94.4)	2 (5.6)	36 (100)
AST/ALT increase	124 (83.8)	24 (16.2)	148 (100)
Asthenia	30 (81.1)	7 (18.9)	37 (100)
Blood bilirubin increase	29 (93.5)	2 (6.5)	31 (100)
Confusion	0	1 (100)	1 (100)
Constipation	8 (100)	0	8 (100)
Dehydration	0	1 (100)	1 (100)
Diarrhoea	114 (89.8)	13 (10.2)	127 (100)
Dizziness	7 (100)	0	7 (100)
Oedema	9 (100)	0	9 (100)
Erythema	5 (100)	0	5 (100)
Fatigue	103 (89.6)	12 (10.4)	115 (100)
Fever	7 (100)	0	7 (100)
H&F syndrome	34 (94.4)	2 (5.6)	36 (100)
Hair depigmentation	11 (100)	0	11 (100)
Headache	17 (100)	0	17 (100)
Hepatotoxicity	8 (53.3)	7 (46.7)	15 (100)
High GGT	5 (100)	0	5 (100)
High LDH	7 (100)	0	7 (100)
Hyperglycaemia	15 (83.3)	3 (16.7)	18 (100)
Hypertension	79 (68.7)	36 (31.3)	115 (100)
Hypertriglyceridaemia	0	1 (100)	1 (100)
Hypocalcaemia	7 (100)	0	7 (100)
Hypokalaemia	1 (50)	1 (50)	2 (100)
Hypomagnesaemia	9 (100)	0	9 (100)
Hypophosphataemia	5 (100)	0	5 (100)
Hyporexia	7 (87.5)	1 (12.5)	8 (100)
INR increase	7 (100)	0	7 (100)
Insomnia	5 (100)	0	5 (100)
Mucositis	37 (100)	0	37 (100)
Nausea	123 (96.1)	5 (3.9)	128 (100)
Pain	19 (90.5)	2 (9.5)	21 (100)
Pancreatitis	0	1	1 (100)
Proteinuria	7 (63.6)	4 (36.4)	11 (100)
Pruritus	4 (100)	0	0 (100)
Rash	18 (94.7)	1 (5.3)	19 (100)
Skin and subcutaneous tissue disorders	6 (100)	0	6 (100)
Skin hypopigmentation	7 (100)	0	7 (100)
Drowsiness	6 (100)	0	6 (100)
Thromboembolic events	0	1 (100)	1 (100)
Urinary tract infection	0	1 (100)	1 (100)
Vomiting	41 (9.1)	4 (8.9)	45 (100)

### Risk of Bias

The studies included in this systematic review were phase II studies. The fact that we included the survival estimates of the pazopanib arm in Bergsland et al.'s study (26) eliminates the potential drawbacks of considering trials with different designs. Similarly, the study by Phan et al. (24) reported distinct median PFS and OS for both pNET and carcinoid tumour arms. We considered these values in the meta-analysis because they came from different studies. The relative similarity between median survival estimates, especially for PFS, partially safeguarded against extreme results.

## Discussion

### Summary of Main Findings

Phase II trials provide a valuable insight into diseases, treatment efficacy and safety, especially in settings where is it difficult to carry out large randomised phase III clinical studies i.e., in the area of rare tumours. In a phase II setting, surrogate endpoints are usually taken into consideration as an early sign of drug activity and can facilitate the decision-making about whether to proceed with phase III testing. Sunitinib is still the only approved TKI for the treatment of advanced pNETs, showing a clear impact in terms of PFS and ORR. However, despite an initial benefit, sunitinib inevitably loses its effectiveness because of the activation of downstream pathways that induce resistance, leading to increased invasiveness and metastasis (27, 28). Peptide radionuclide receptor therapy (PRRT), chemotherapy and everolimus are other therapeutic options, but patients progressing on these treatments are left with few, if any, alternatives (29).

To the best of our knowledge, the present systematic review is the first to assess phase II literature on the effectiveness of pazopanib in NEN. Pazopanib achieved a DCR of 91.3% and a median PFS and OS of 11.6 and 24.6 months, respectively, superior to results of other targeted therapies in the same setting (DCR ranging from 72 to 84% and median PFS of 11–12.6 months) (12, 30–32). Of note, although half of the patients were pretreated, the pazopanib activity was maintained. Furthermore, the addition of SSAs would appear to promote a synergistic effect, increasing the DCR in this patient subgroup. A recently published network meta-analysis supports this hypothesis of the additional effect of the SSA combination with other therapies (33).

Recently, some phase II trials have been carried out to obtain a breakthrough therapy designation from the regulatory authorities for tumours whose therapeutic armamentarium is limited (34, 35). However, the interpretation of data from phase II trials has faced difficulties because of the lack of a control group, hampering direct and scientifically robust comparisons, and small patient samples. The added value of a phase II systematic review and meta-analysis could help to overcome the problem of sample size for patients treated in single trials and amplify the efficacy data of a drug evaluated prospectively in small studies.

Safety profile is also crucial factor. The results of the present review indicate that pazopanib carries a substantial risk of adverse events that can affect patient quality of life. However, the incidence of G3-G4 toxicities reported in the largest and most recent trial was 15% lower than that of previous studies. These data suggest an increasing familiarity with pazopanib over time due to its ł widespread use, and a better management of it side-effects. Overall, given that pazopanib seems to have a disease control rather than curative effect in NENs, quality of life should be take in consideration in future prospective studies.

### Limitations

This study has some limitations. We conducted a comprehensive literature search with a sensitive search algorithm and an extensive manual search of reference lists and conference proceedings. However, we were unable to obtain additional unpublished data and are aware that a substantial amount of information is not available to the public. Another limitation is the low number of phase II clinical trials with different types of study design and populations included. Despite this, we believe that our results could provide important indications for the design of future dedicated clinical trials on NETs to underline the importance of head-to-head comparisons and the correct patient setting. Furthermore, the addition of SSAs to experimental drugs could be taken into consideration when designing dedicated trials on NETs.

## Conclusions

Overall, our current pooled analyses of data on pazopanib in phase II studies are essentially consistent with the data available for other approved drugs. Surprisingly, although pazopanib was one of the first and most widely studied TKIs in neuroendocrine tumours, it has not moved to phase III. For this reason and because of the rarity of the disease, we decided to further investigate pazopanib activity in terms of DCR and mPFS. The clinical information available supports the use of pazopanib for the treatment of metastatic neuroendocrine tumours of different origin, especially those of the gastrointestinal tract.

## Data Availability Statement

The datasets generated for this study are available on request to the corresponding author.

## Author Contributions

AB, CL, and TI conceived the idea for the study and drafted the article. GD, VF, CC, and FR were responsible for data acquisition. SN, SS, AV, and AD performed the meta-analysis and co-drafted the manuscript. CS, GM, and LM assessed the quality of the manuscript independently through the Downs and Black checklist. All authors read and approved the present version of the paper for submission.

## Acknowledgements

The authors thank Gráinne Tierney and Cristiano Verna for editorial assistance.

### Conflict of Interest

The authors declare that the research was conducted in the absence of any commercial or financial relationships that could be construed as a potential conflict of interest.
